# What's law got to do with it part 1: A framework for obesity prevention

**DOI:** 10.1186/1743-8462-5-10

**Published:** 2008-06-05

**Authors:** Roger S Magnusson

**Affiliations:** 1 Faculty of Law, University of Sydney, Sydney, Australia

## Abstract

This article provides a conceptual framework for thinking about the role of law in responding to population weight gain in Australia. Part 1 focuses on two core questions. Firstly, in pursuing the aim of weight reduction at the population level, what should law be trying to influence? The challenge here is to identify a model of the determinants of obesity that is adequate for legal purposes and that illustrates the entry points where law could best be used as an instrument of public health policy. Secondly, what kinds of strategies and tools can law offer to obesity prevention? The challenge here is to identify a model of law that captures the variety of contributions law is capable of making, at different levels of government, and across different legal systems.

In Part 1 of the article, I argue that although law can intervene at a number of levels, the most important opportunities lie in seeking to influence the social, economic and environmental influences that shape patterns of eating and nutrition across the population as a whole. Only policies that impact broadly across the population can be expected to influence the weight distribution curve that has shifted relentlessly to the right in recent decades. Part 2 of the article builds on this analysis by offering a critical review of selected legal strategies for healthier nutrition and obesity prevention.

## Background

The rapid rise in rates of overweight and obesity among Australian adults and children has intensified debate about the most effective and appropriate strategies for obesity prevention. Law's role in these efforts is still evolving and remains heavily contested [1-4]. There are many opportunities for scare-mongering and political stunts. One food industry lobbyist, for example, warns that the war on obesity could lead to "laws that would let a waiter decide if a patron could order dessert" [5]. On the other hand, in Mississippi, the most obese state in the United States, a recent Bill went so far as to prohibit restaurants from serving obese people [6]. The chances of this proposal ever becoming law were risible, and it was predictably thrown out by a House subcommittee [7]. While legal absurdities like these should be disregarded, the link between obesity and the over-consumption of high-fat and high-sugar foods has created a public relations crisis for the processed food industry. With large profits at stake, there is a struggle for regulatory control.

Beyond the economic contest, obesity has become a crucible for debating the appropriate role for government, and for law, in public health generally. Some commentators frame the causes of – and the solutions to – obesity in purely individualistic terms. On one view, obesity is neither a contagious disease, nor a pervasive hazard (like pollution) that creates significant externalities and demands a collective response [8,9]. Government involvement is treated with suspicion, as "a giant government land grab" [8] or as nanny-state interference that ignores "personal responsibility".

Public health advocates, for their part, tend to regard patterns of eating and physical activity within the population as the outcome of a broader and more complex system of determinants, many of which are outside the control of individuals [10]. A public health approach asserts that responsibility for obesity and lifestyle health risks is shared more widely, and that in view of *their *responsibility for the public's health, governments should recognise the systemic constraints on behaviour and act assertively to make healthy lifestyles easier [11,12].

Public health advocates have resisted industry attempts to narrow the obesity debate to issues of energy expenditure alone ("kids watch too much TV and aren't getting enough exercise"). Energy intake issues are important priorities for policy [13], and increases in energy consumption appear to be the major factor driving the trend towards population weight gain [13-17]. No one doubts that personal responsibility has an important place in public health policy. But public health advocates are mindful that over-emphasising individual responsibility could absolve the food industry from responsibility for its manufacturing and marketing practices, while condemning health policy to (yet) another round of the motivational initiatives that have so far failed to halt the trend.

Political rhetoric about obesity – possibly reflecting public attitudes – has struggled to move beyond the individualistic approach. In Australia, the philosophical stance of the former Howard government was best summarised by its Minister for Health and Ageing, Tony Abbott MHR, who said: "In the end, our weight is largely a product of the amount of exercise we do and the amount of food we put in our mouths. And obviously we are in almost total control of both of those issues" [18]. In March 2008, the House of Representatives Standing Committee on Health and Ageing announced an inquiry into the impact of obesity in Australia [19]. It remains to be seen whether a more sophisticated understanding of the forces driving changes in population weight patterns will emerge from this inquiry.

Media coverage of obesity on Australian television has tended to frame the issue as an individual problem caused by poor personal nutrition, rather than in terms of system-wide factors contributing to a population-wide trend [20]. Not surprisingly, this contributes to the assumption that if law were to become involved, it would mean militaristic, jackboot-style interventions that would trample on personal and economic freedom, while ignoring personal responsibility. General community attitudes to regulation are perhaps best summarised by a Scottish journalist who writes: "The state can offer advice and can lean heavily on the food manufacturers, but it has no right to interfere in the diet of adults, unless they are cannibals" [21].

Part 1 of this article offers a framework for conceptualising the roles that law could play in obesity prevention at the population level in Australia. A population health approach is less focused on strategies for weight loss in individuals, and more interested in strategies for reducing energy intake and encouraging physical activity across the population as a whole. Only policies that impact broadly across the population can be expected to influence the weight distribution curve that has shifted relentlessly to the right in recent decades.

The role that law plays in policy efforts to address obesity will differ according to circumstances unique to each country. In the United States, for example, legislation has been enacted in order to:

○ establish commissions of inquiry into obesity;

○ detail how monies shall be spent on particular programs;

○ set out exactly what a specific program, such as a childhood obesity prevention program, shall do [22];

○ specify the content of the school curriculum [23]; and

○ establish "Obesity Awareness Week" and "Fruit and Vegetable Month" [24].

In Australia, separate legislation is not necessarily required to achieve these aims, and although program spending decisions are ultimately reflected in budget legislation, the role of legislation in responding to obesity goes well beyond this (as part 2 of this article will illustrate).

Despite great interest in obesity-related litigation, lawsuits against specific food and beverage manufacturers for harm caused to obese plaintiffs seem even less likely to succeed than tobacco lawsuits [1,2,25,26]. A comprehensive approach to obesity prevention requires policies that engage with a range of determinants extending well beyond food composition and marketing practices (the likely targets of any lawsuits). This article focuses on legislation and executive action, rather than litigation.

After briefly considering obesity statistics in Australia, part 1 of this article will address two key questions. Firstly, in pursuing the aim of obesity prevention at the population level, what should law be trying to influence? The challenge here is to identify a model of the determinants of obesity that is adequate for legal purposes and which illustrates the entry points where health policies might take *legal form*. Secondly, what kinds of interventions and strategies can law contribute to obesity prevention? The challenge here is to identify a model of law that captures the variety of contributions law is capable of making, at different levels of government, and across different legal systems. Part 2 of this article will consider a basket of laws and interventions that could – if implemented – plausibly constitute a "law of obesity" for Australia.

## A snapshot of obesity in Australia

Overweight and obesity are among the leading risk factors contributing to the burden of disease in Australia, accounting for 7.5% of years of life lost due to disability and premature mortality in 2003 ([27], pp72-75). This burden includes the contributions that obesity makes to hypertension, raised cholesterol, and impaired glucose tolerance, and to a range of chronic conditions including coronary heart disease, stroke, various cancers (including colon and breast) [28], type II diabetes, gallbladder disease, osteoarthritis, gout, and sleep apnoea [29].

The prevalence of overweight and obesity is based on measurements of the Body Mass Index (BMI), which divides weight in kilograms by height squared (m^2^). Self-reported data from the 2004–05 National Health Survey show that 19% of men and 17% of women (2.5 million Australians) were obese (BMI ≥30 kg/m^2^). A further 41% of men and 25% of women (4.9 million) were overweight (BMI 25.0–29.9 kg/m^2^) [[30], p183]. Data based on measured height and weight in the AusDiab study carried out in 1999–2000 showed higher rates: 19% of men and 22% of women were obese, with a further 48% of men, and 30% of women overweight [31]. Obesity prevalence showed a 2.5-fold increase between 1980–2000 [31]. In New South Wales, rates of overweight and obesity among school-age children have climbed from 11% in 1985, through 20% in 1997, to 25% in 2004 [32]. The lack of continuous nutritional surveillance is a constraint for policy-makers in Australia [33,34]. Good health data is an important stimulus for action: national nutrition surveys should measure weight, range and frequency of physical activity, and the mix of foods consumed. Linking results to local government area would enable programs and funding to be appropriately targeted to areas of greatest need.

Weight gain in the Australian population is consistent with trends in other industrialised countries. In the United States, the prevalence of obesity remained relatively stable from 1960–80 but climbed sharply thereafter, doubling in adults from 15% (1976–80) to 32.3% in 2003–04 [35,36]. Over the same period, overweight in adolescents aged 12–19 (defined as BMI for age at the 95^th ^percentile or higher) tripled from 5% to 17.4% [36,37]. In 2003–04, 66.3% of adults were overweight or obese, while 33.6% of American children and adolescents were either overweight, or at risk of overweight (BMI at 85–95 percentile for age) [36]. In England, 65% of men and 56% of women were either overweight or obese in 2003 [38]. The rate for children aged 2–15 was 32% for boys and 31% for girls [38].

## What is law trying to influence? Law and the determinants of obesity

When it intervenes in support of obesity prevention, who or what should be the "target" of law's interventions? One way to clarify this question is by asking: who gets fat? Is it cells? Is it individuals? Is it target groups in the population, or, indeed, the whole population? [cf [39], pp28–29]. Each of these different levels offers a distinct perspective – and different policy prescriptions – on the problem of obesity.

**Figure 1:**
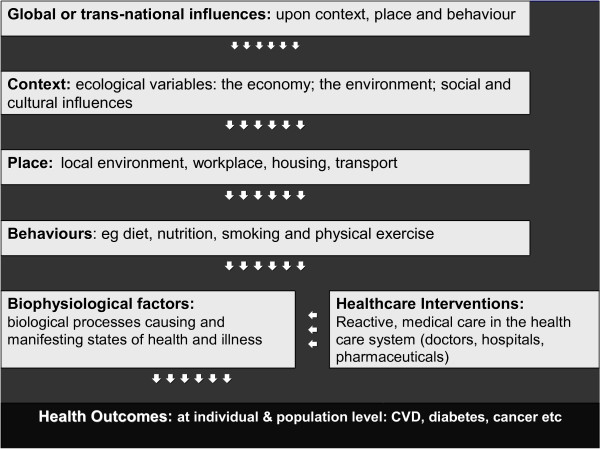
A simplified hierarchical model of the determinants of health and disease.

According to the "determinants of health" model that is widely used in public health, health and illness can be understood as the product of influences that cascade from the global level, down to the level of our genes. This hierarchy of determinants is presented in many different ways in order to highlight particular influences or associations. In Figure 1, health determinants are presented in terms of a hierarchy ranging from *global and macro factors *(economic, environmental, social and cultural factors), through *mid-level factors *(local environment, workplace, housing), to *proximate factors *including individual behaviours and lifestyles. Together, these categories of determinants interact with the genetic and biological characteristics of individuals and populations and with medical interventions within the health care system, to produce "health outcomes" at the individual and population level [[30], pp141-146]. Determinants can impact on health either negatively, as risk factors, or positively, as protective factors. Some determinants are not modifiable (such as genetics, age, sex, and basic metabolic rate), whereas others are modifiable to a greater or lesser extent (for example, smoking, diet, physical activity and education).

The twentieth century reflected a gradual broadening of the determinants that are understood to contribute to states of health and illness in the population, and a growing realisation of their complexity and inter-relationships. Although obesity and chronic diseases are often seen in terms of the proximate, behavioural risk factors that provide the immediate explanation for these outcomes, a determinants of health approach requires one to work outwards towards other frames of reference (the economy, the workplace, the transport system, the media, the local environment), since what happens in these arenas influences how people live, work, eat and play.

### The determinants of obesity and corresponding policy interventions

**Figure 2:**
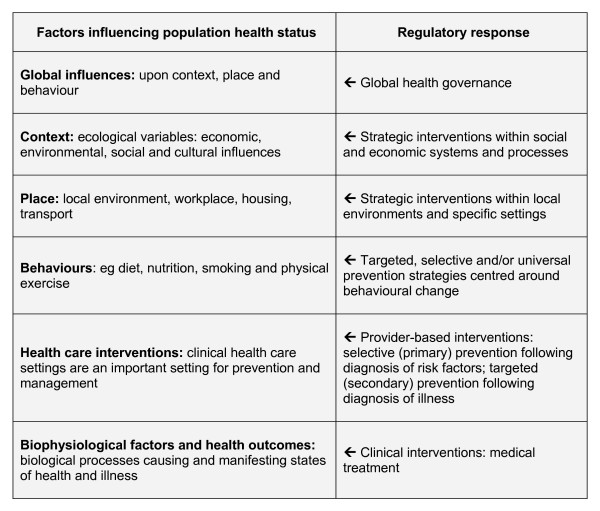
A simplified hierarchical model of the influences upon health, and corresponding policy interventions*. *This figure was partly adapted from J. McKinlay and L. Marceau, "A Tale of 3 Tails," *American Journal of Public Health *89 (1999): 295–298, at 296.

As Figure 2 illustrates, it is possible to identify policy initiatives for obesity prevention that correspond to each level of the hierarchy of determinants. In fact, the determinants of health model creates a kind of "geography" for locating where policy attention *is *being directed at Commonwealth and State level, and the likely strengths and weaknesses of those approaches in achieving their intended outcomes.

### The medical model: provider-based prevention

A "medical model" would respond to obesity in a reactive fashion by treating the physical manifestations of obesity-related illness, including with bariatric surgery in the severest cases [40]. Although clinical medicine seeks to mitigate the effects of chronic disease, the clinical encounter also provides opportunities for assessing patients and assisting them to quit smoking, to lose weight and to address other risk factors for chronic disease. Continued smoking, for example, makes a wide range of chronic diseases worse, increasing the risks and speed of disease progression and of death [41]. There is a growing body of literature that advocates models for intervention and explores how to overcome the financial, time, knowledge and ideological barriers that prevent the integration of health promotion and disease prevention strategies within clinical care encounters [42-47]. Building prevention into primary care is an important priority in future. Law could support this process by facilitating the extension of private health cover for screening and preventive services [48], or by creating incentives for health professionals to carry out prevention-focused activities in primary care, and as part of employer-funded health and wellbeing programs.

### The behavioural model: incentives for altering modifiable lifestyle risks

A "behavioural model" – both within and beyond health care settings – would focus on modifiable lifestyle risks, seeking to educate and to create incentives for behavioural change. These health promotion strategies could be framed as targeted prevention efforts that would engage with already-overweight individuals in community settings, or with those at risk of becoming obese, or with those who have other risk factors for chronic disease. Universal prevention initiatives would seek to educate, motivate and support healthy lifestyles within the community generally [[49], pp159-162]. Australia's public health care system (Medicare) is taxpayer-funded, and available to all irrespective of the lifestyle-related health risks of each individual. Similarly, private health insurance is community rated [50]. It is conceivable, however, that law could re-assess this approach and seek to create incentives for reducing future health risks and expenditures through behavioural change; for example, through health insurance discounts for individuals who could demonstrate that they had taken steps to reduce excess weight, to improve their diet, or to quit smoking.

There is no doubt that behavioural changes including weight control, reduced intake of saturated fats, more physical activity, and smoking cessation would make an enormous difference to the national burden of chronic disease *if only *they were implemented in lifestyles across the nation [51,52]. However, as the trend towards obesity, diabetes and other chronic conditions illustrates, many people find it difficult to maintain a stable weight and to implement healthy behaviours [53-55]. This is unlikely to change so long as the environmental factors that subtly influence the daily decisions that lead to energy imbalance and weight gain remain unaddressed.

There is evidence that lifestyle intervention programs for high risk groups (typically recruited in medical settings), can reduce risk factors and progression to chronic disease [56,57]. Although such programs are part of a comprehensive policy package, they tend to be time-consuming and therefore expensive to run. They also focus on relatively small numbers of people and require each participant to become, in effect, a patient. It is not clear that such programs could be cost-effectively rolled out on a population-wide basis [58,59]. At any one time, a large proportion of the community are either attempting to lose weight or to maintain weight by various forms of dieting and lifestyle changes. These attempts are mostly unsuccessful [60], and this is reflected in the steady rise in obesity rates. It seems highly doubtful that community education and motivational campaigns, as stand-alone strategies, will be successful in unlocking new personal resources not already being tapped by the weight loss industry or by individuals themselves. So far, none of these strategies has been effective in reversing the trend.

### The ecological model: addressing influences upon patterns of behaviour

**Figure 3:**
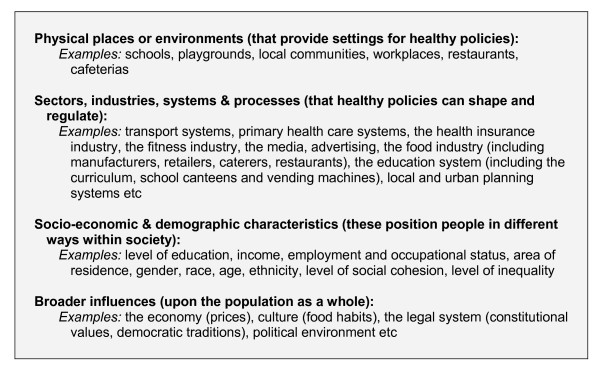
A model of the ecological determinants of health encompassing environmental and socio-economic factors.

As policy moves up to engage with the environmental and socioeconomic determinants that influence the context, and the specific local conditions within which lifestyles are lived, the policy focus changes from behaviour per se, to the forces and processes that channel and create *patterns of behaviour*. Figure 3 provides one way of conceptualising the influences that occur at the level of *place *and *context*. It conceptualises these "ecological" or structural influences in terms of four intersecting categories: (i) physical environments or places; (ii) sectors, industries, processes and systems; (iii) socio-economic and demographic characteristics; and (iv) broader influences. Policies that engage with physical environments, sectors and industries, and the social, cultural and economic factors that broadly define the national context, have the capacity to influence the behaviour and average health status of broad populations.

Because "social and economic conditions and physical environments are [largely] created by sectors other than health", an ecological approach implies interventions that range well beyond the medical and health sector [61]. A "whole of government" approach is required to coordinate the activities and to mediate the conflicting interests of different sectors, government departments, and functionally separate agencies. An ecological policy approach is, by nature, complex: a portfolio of interventions will be required to successfully address the constraints that an obesogenic environment places on healthy lifestyle choices [62]. Environmental interventions may affect a wide range of people, including those who do not understand themselves to be at risk and do not appreciate the "interference" ([63], pp12-13). At the same time, any single policy – such as a tax on high-fat snack foods, or a ban on junk food advertising – can only be expected to make a modest contribution to the overall goal. Ecological approaches may also raise controversy when they interfere with powerful economic interests, particularly in the food sector.

### The importance of an ecological approach

Despite this, there are important reasons why a policy framework that emphasises policies at the ecological level is likely to provide a more successful and sustainable response to population weight gain. Firstly, over 50% of the population is already overweight or obese. Dieting by individuals is common but has so far proven ineffective in reversing population trends [[49], pp179-194]. The rationale for a population-based approach is to address the underlying, environmental risk factors that impact on the entire population and make obesity increasingly common. Such an approach could boost the effectiveness of both provider-based and community-based prevention. By focusing on creating supportive policy environments that encourage healthy lifestyles, it becomes "less necessary to keep on persuading individuals" [64].

Secondly, the classification of normal weight, overweight and obesity into specific bands is arbitrary. The risk of diabetes and heart disease rises modestly with increases in BMI within the normal range, but exponentially at higher levels [65]. Cases of chronic disease do not only arise among those whose lifestyle risk factors (overweight, high blood pressure, high cholesterol and sedentary lifestyle) place them on the extreme right hand tail of some distribution, but also "from the many people in the middle part of the distribution who are exposed to a small risk" [66]. The size of the health threat posed by "widespread inconspicuous risks" is easy to overlook when the absolute risk of the population – as reflected in its weight distribution curve, for example – is already too high as a result of environmental factors to which the population as a whole is exposed [[67], p38]. Small changes in average weight could make a significant difference, but this requires policies capable of engaging with the population as a whole.

Thirdly, obesity is a social justice issue. A socioeconomic gradient exists for chronic illness and associated risk factors in Australia [54,68,69], including obesity and quality of diet. The prevalence of obesity has risen for all socioeconomic groups, but a socioeconomic gradient remains and is particularly pronounced as measured by level of education, income quintile, and occupation [68]. There is a graded association between obesity and relative income disadvantage at the local area level, for both men and women [68,70]. King points out that the right-shifted population distribution of BMI in the most disadvantaged census districts may account for significant differences in the burden of disease between most and least disadvantaged areas [70]. In rural Australia, rates of overweight and obesity for men and women are particularly high [54,71], casting doubt on the hypothesis that features of the urban environment are driving the obesity epidemic, to the exclusion of energy intake issues. Studies also confirm that those with low levels of education and in low-income households are less likely to purchase foods high in fibre and lower in sugar, fat and salt [72,73]. All of this evidence suggests that, if policies are to successfully redress health inequalities, they must be broadly based, while also engaging with local environments.

To summarise so far: there are two distinct approaches competing for influence in obesity policy. One view, strongly supported by public health experts, argues that socio-economic and environmental factors have created an obesogenic environment that creates risks for the entire population, as reflected in sedentary lifestyles, unhealthy eating patterns, and increasing rates of overweight and obesity [74]. The other view, which tends to reflect popular assumptions about obesity, focuses more on personal responsibility and self-control. The latter view suggests that policy efforts should be directed towards education and encouraging individuals to live healthier lifestyles. On the other hand, unless policies also seek to address the ecological causes of weight gain, there is little to prevent the occurrence of new cases [[49], p178].

### The challenges of an ecological approach

An ecological approach to population weight gain brings its own challenges. For those with a vested interest in the status quo, it is tempting to downplay the significance of those factors that collide with one's self-interest, while pointing to other factors as the "real" culprits. In one recent example, a franchisee of nine McDonald's restaurants (and an aspiring politician) told reporters that there were many reasons other than fast food for "the propensity of kids to be bigger now than they were when I was growing up". "It's to do with lifestyles, kids in front of television and computer screens, kids being driven to school and picked up rather than riding their bicycles" [75]. While no one doubts that many factors are involved, including changing patterns of physical activity, the challenge for an ecological approach is to ensure that food and nutrition are not *excluded *from the policy agenda, merely because this suits commercial interests [76]. An ecological basis provides a basis for identifying a range of factors that can plausibly be linked with population weight gain, and a corresponding basket of "plausible interventions" whose combined weight could make a difference. Serious investment in obesity prevention policies is needed now, before things get worse, relying on the best available evidence, rather than perfect information [58,74,77].

Despite this, it is neither possible nor desirable to address all of the factors that have played a role in population weight gain. Some factors, such as the participation of women in the workforce, or the availability of ready-made food, can be regarded as highly desirable. It follows that the solutions to obesity may not necessarily revolve around the factors that have given rise to the problem in the first place. As Professor Gregg Bloche points out, "Not all causes imply viable remedies. And conversely, effective remedies...need not operate via the causal pathways that explain obesity's epidemic surge" [[78], p1338].

## What kinds of tools can law offer to obesity prevention?

Public health lawyers and public health professionals typically approach the links between law and patterns of health eating and physical activity from different directions. The latter have tended to scan the policy environment, identifying settings where new laws might play a helpful role [10,12,79]. Sacks, Swinburn and Lawrence extend this approach by identifying the differing responsibilities and opportunities for local, state and federal governments to develop policies across the food system and physical activity environments [80].

Public health lawyers, on the other hand, have tended to begin with the law itself, categorising different forms of legal intervention, as distinct from the key sectors or policy settings where these laws would operate. Professor Lawrence Gostin's widely influential model categorises the law's impact upon the public's health under seven headings. These include:

○ the power of government to tax and to spend;

○ the power to alter the informational environment;

○ the power to design and alter the built environment; and

○ the power to respond to health inequalities by addressing socio-economic disparities.

Gostin points out that governments can regulate persons, professionals and businesses in a direct and prescriptive fashion, and that private actors and governments can indirectly influence public health outcomes by pursuing legal claims through the courts. Where law itself is a barrier to health, deregulation may be an effective strategy [81].

**Figure 4:**
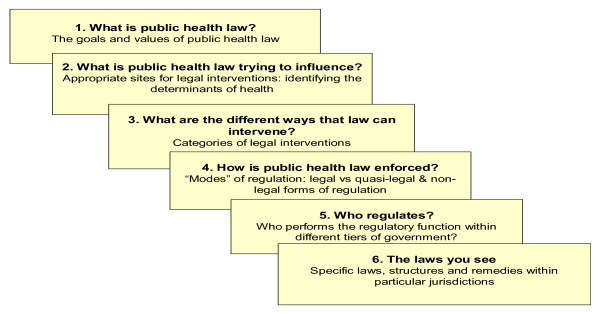
A framework for thinking about law and public health.

Elsewhere, I have proposed a model for understanding the links between law and public health in a way that brings the perspectives of lawyers and public health advocates closer together. The model presents law's engagement with chronic disease in terms of six conceptual frames (see Figure 4) [82].

### Values

The first frame involves consideration of the broad values that society brings to its public health laws. This includes the extent to which society *values *improvements in its collective health, relative to other collective goals, as well as the weight to be given to collective goals relative to personal and economic freedoms. Debate about underlying values is often the real issue that motivates the question: "what is public health law?" The boundaries of law's role in obesity prevention will inevitably be drawn by value judgments about what is the business of law, and what is not. These values and boundaries should be recognised and debated.

### Targets or settings for policy

The second frame directs attention to the hierarchy of determinants and the priority settings or targets for legal interventions. As argued above, if the aim is to stabilise and reverse population weight gain, then law must engage with the factors that do, in fact, influence the *average behaviour *of the population. This means that law should go beyond targeting high risk groups and focusing narrowly on their behaviour. Targeted interventions are vital for responding to health inequalities and for directing resources to the most disadvantaged. At the same time, law needs to focus on the economic, environmental and social factors that shape *patterns of behaviour *within the population.

### Legal tools

**Figure 5:**
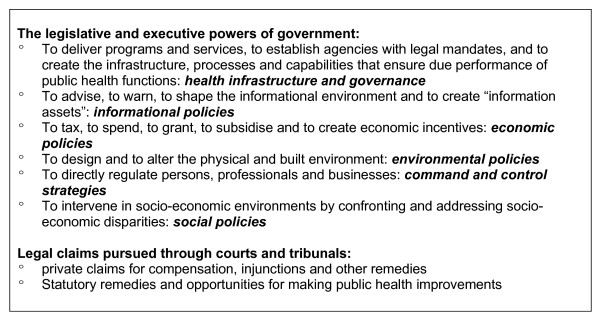
**A typology of legal interventions for public health improvement**. *This figure is heavily indebted to Lawrence O. Gostin, "Law and Ethics in Population Health" *Australian and New Zealand Journal of Public Health *2004, **28: **7–12. It also draws on Wendy C. Perdue, George A. Mensah, Richard A. Goodman, Anthony D. Moulton, "A Legal Framework for Preventing Cardiovascular Diseases" *American Journal of Preventive Medicine *2005, **29(5S1): **139–145.

The third frame directs attention to how law will intervene within particular sectors and policy settings in order to address population weight gain. Here, the model builds on the work of Gostin and other scholars who have sought to categorise the various strategies law can adopt in chronic diseases prevention [[81,83]; Figure 5]. Law is, of course, a determinant of health in its own right. As Figure 5 illustrates, legislative and executive powers can be used to implement economic policies, to shape the informational environment, to shape the physical environment, to give effect to social policies, and to otherwise impose legal requirements on individuals and businesses in a direct, prescriptive manner. In addition to regulating the external environment, legislation and executive actions can seek to improve the coherency of the government's own regulatory functions; for example, by creating agencies or inter-agency or inter-governmental processes for attacking complex problems. A persistent impediment to a comprehensive approach to obesity prevention is the lack of effective governance structures for ensuring a coordinated, multi-sectoral approach to policy-making in this area.

### Mechanisms for implementation and enforcement

The fourth frame overlaps with the third, directing attention to the mechanisms for implementation, compliance and enforcement of policies for obesity prevention. Public health law has typically relied upon prescriptive, "command and control" methods that impose specific constraints and standards of conduct upon individuals and businesses, enforceable through civil remedies, criminal penalties and licensing requirements [[83], p140]. Performance-based regulation, on the other hand, as advocated by Sugarman and Sandman [84,85], would involve governments fixing food and beverage companies with responsibility for achieving specific targets for reduced rates of obesity among children, while leaving these companies free to determine how these should be achieved. Other mechanisms for ensuring compliance rely on economic pressure, education and the provision of information, self-regulation, and market dynamics. The tension between the behavioural and ecological approaches to obesity policy has its parallel in the tension between voluntary and regulatory approaches to policy implementation.

### Level of regulation

The fifth frame directs attention to the issue of *which level of government *has constitutional or legislative power to regulate. In the United States, federal pre-emption provisions have been used to "federalise" control of important public health issues, such as tobacco advertising, and cigarette warnings labels. Conditional grants to state and local government bodies are also a pronounced feature of federal law-making that has enabled the federal government to impose policy on a national basis. Despite this, a robust tradition of law-making at the state and local/city level has been responsible for many of the innovative responses to tobacco and obesity in the United States. Mechanisms for strategically relaxing control, imposing control, and for claiming regulatory control, at each level, provide important opportunities for enhancing the reach of public health regulation in federal systems.

The frames set out above provide a conceptual approach for systematically unpacking law's relationship with public health, including obesity and chronic disease, at national and sub-national levels. The final frame (and the sum of each of the preceding ones) is the specific laws that exist, or that one advocates, for addressing a public health issue within a particular jurisdiction.

At its simplest, the "law of obesity prevention" lies in choosing from the variety of tools and strategies that law has on offer, and matching them to the sectors and settings where interventions are most needed. There are many ways that this can be done, and many ways of categorising the results. Part 2 of this article will review some of the most important legal interventions that have been advocated, categorised according to the underlying legal strategy that they represent.

## Conclusion

While some have advocated a "breakthrough change" approach to reversing population weight gain [86,87], the reality is that change is more likely to be incremental [88]. Regulatory approaches to obesity prevention roughly resemble tobacco control efforts thirty years ago. Policy-makers are sceptical about the merits of legal and regulatory interventions, although this is changing subtlety, with due regard to the differences between tobacco and food.

For many people, "personal responsibility" is a deeply entrenched idea that represents the only plausible solution to population weight gain. Health policy-makers, on the other hand, would be wise to resist the temptation to treat populations as if they were no more than a collection of individuals. Obesity prevention with individuals necessarily means encouraging a healthy lifestyle by maintaining a healthy diet, minimising sugar, salt and saturated fats, eating plenty of fruit and vegetables, and exercising faithfully. The fact is, however, that only a small fraction of the population actually manage to do all these things [89,90]. Some 60% of men, and 42% of women, are either overweight or obese [30]. Fortunately, for populations, other kinds of preventive interventions are also possible. In addition to education and health promotion, ecological policies that impact on broad segments of the population and are capable of altering average patterns of behaviour, will be required if we hope to influence the weight distribution curve that has shifted relentlessly to the right in recent decades.
